# Evolutionary patterns and microscopic mechanisms of strength in mine tailings backfilled with waste glass

**DOI:** 10.1038/s41598-023-50807-9

**Published:** 2024-01-03

**Authors:** Daiqiang Deng, Yu Gao, Zimin Chen, Ye Wang

**Affiliations:** College of Civil Engineering, Xiangtan University, Xiangtan, 411105 China

**Keywords:** Engineering, Materials science

## Abstract

In order to promote the sustainable use of resources and reduce the waste of waste glass and tailings resources. The present study focuses on a fluorite mine as the research subject, utilizing coarse tailings, fine tailings, cement substitute-curing agent, and recycled waste glass as the primary raw materials. It investigates the changes in compressive strength of coarse tailing with varying sand- binder ratios and glass content at 3-day, 7-day, and 28-day intervals when the filling slurry concentration is set at 77% and the ratio of coarse tailings to fine tailings is maintained at 2:1. The findings indicate that there is minimal impact on the compressive strength of test blocks when using a sand binder ratio of 4:1 and a glass sand content below 10%. However, once the glass sand content exceeds 10%, a significant decline in compressive strength occurs. Scanning electron microscope (SEM) images reveal ettringite crystal formation in test blocks with both 0% and 25% glass sand content due to high levels of Na_2_O in the glass sand. This leads to internal expansion within test blocks resulting in reduced strength. Notably, when using a sand-binder ratio of 8:1 along with a glass sand content of 25%, early strength characteristics are observed for test blocks. Furthermore, incorporating glass sand has little influence on late-stage strength for backfill when employing either an 8:1 or 12:1 sand-binder ratio. Based on this experiment conducted under conditions including mass concentration of 77%, the optimal waste-glass-to-mine-tailings-filling-sand-binder-ratio is determined as 8:1with a corresponding glass content of 25%.

## Introduction

The disposal of waste glass, a prevalent form of solid waste in daily life, has been confronted with significant challenges. The current treatment of waste glass in the field of environmental protection primarily involves recycling or landfilling. However, landfilling consumes significant land resources and gives rise to environmental issues^1–3^. The utilization of waste glass as a backfill material for mine tailings not only effectively addresses the issue of waste glass, but also enhances the stability and plasticity of tailings backfill, thereby reducing mining activities and environmental footprint while achieving the dual objectives of resource recovery and environmental protection^4–7^.

Mine tailings backfill is a commonly used treatment method in mines. It involves the process of backfilling the tailings generated during mine beneficiation into the underground mining area. This method serves the dual purpose of managing the tailing waste and significantly reducing the pollution caused by harmful elements to the environment. Additionally, it helps in controlling the subsidence of the mining area's surface and addresses other related issues^8–11^. The management of the environment and the goal of resource recovery can be achieved mainly through the use of loose and semi-dry tailings. These tailings are used to fill the voids created by the mining process, ensuring the recycling of residual ore and maintaining environmental sustainability^12–15^.

Numerous scholars have conducted extensive research on the utilization of waste glass. Liu et al.^16^ conducted an orthogonal test, substituting cement with glass powder and river sand with glass sand. The results were analyzed using polar analysis and analysis of variance. The optimal glue and sand ratio of 1:4 was obtained with a mass concentration of 84%. The dosage of glass powder was 65%, and the dosage of glass sand was 50%.

Zhenbang et al.^17^ in order to investigate the effect of superplasticizer (SP) on the flowability of ultrafine tailing sand cemented filler (UTCPB). The water film thickness (WFT) theory was introduced to investigate the flowability of UTCPB doped with superplasticizers, and the unitary function of WFT was used to characterize the flow extension of UTCPB, and the results of the study helped to further understand the effect of SP on the flowability of UTCPB.

Tugrul et al.^18^ considered sand as a copper tailings replacement for collodion mine filling and captured the effect of sand as tailings replacement on CMF properties by destructive and non-destructive methods. The contribution of ultrasonic non-destructive testing as an alternative method is presented, as well as the impact of their linkage on backfilling techniques.

Muhammet et al.^19^ used different proportions of sand as a partial replacement of tailings in the replacement cemented filling body (CPB) and investigated the long term aging properties of CPB including but not limited to mini slump, mechanical, microstructural, SEM. Finally, it is concluded that sand improves the various properties of CPB and provides for sustainable mining or filling operations.

Bingwen et al.^20^ proposed a non-polluting alternative technology, microbial induced calcite precipitation (MICP), as a promising solution for sustainable fillings.

Yafei et al.^21^ investigated the mechanism of the effect of mixing time on the properties of nanometer SiO_2_ (NS) doped ultrafine tailings collodion filler (SCPB) by mechanical test, slump test, rheological test and microscopic test. It was shown that appropriate NS doping could play a catalytic role to promote gypsum hydrolysis and increase the generation of hydration products.The addition of NS led to the formation of a more structurally stable C–S–H gel with lower Ca/Si and H_2_O/Si and longer average silica chain lengths, which improved the UCS of SCPB.

Zhao et al.^22^ examined the impact of using waste cathode ray tube glass sand on mortar properties. The findings revealed that as the percentage of waste cathode ray tube glass sand replacement increased, both the ASR expansion value of mortar and the metal lead leaching value of hardened mortar also increased. However, these values remained within the safe range. Moreover, the study found that incorporating mineral admixture in the waste cathode ray tube glass sand mortar enabled its use as a recycled material for construction mortar in dry environments.

Amar et al.^23^ conducted a study to investigate the impact of waste glass powder dosage on the fresh and hardened properties of self-compacting repair mortar mixes. The findings revealed that the addition of glass powder had an adverse effect on the workability of the mortar. However, in the hardened state, when the waste glass powder dosage did not exceed 20%, it resulted in improved strength development, durability, and adhesion to the substrate of the mortar after 28 days. In a similar study, Kim et al.^24^ incorporated finely ground waste glass into fly ash and ground slag-based alkali-inspired mortar as a replacement for natural sand. The researchers examined the mechanical properties, water absorption, apparent porosity, and durability of the synthetic mortar. Their findings demonstrated that the efficiency of finely ground waste glass varied depending on the binder ratio used in the synthetic mortar.

Bouachera et al.^25^ conducted a study on the combination of sludge, glass waste, and clay in lightweight aggregates. They prepared these aggregates using ternary and binary formulations, and subjected them to high temperature treatment. The results revealed that the incorporation of glass waste resulted in the formation of a vitreous phase on the surface of the agglomerates. This, in turn, enhanced the physic-mechanical properties of the agglomerates. In another study, Kazmi et al.^26^ utilized crushed waste glass as a substitute for conventional sand. The shear strength test indicated that the friction angle of crushed waste glass under saturated conditions was higher compared to that under dry conditions. These findings suggest that crushed waste glass exhibits improved stability when saturated.

Guo et al.^27^ conducted a study on the water permeability, consistency, and density of construction mortar using varying proportions of glass sand as fine aggregate. The research findings revealed that the permeability of mortar increased as the content of glass sand increased, reaching its peak value at approximately 60–80% doping of glass sand. Additionally, the consistency and density of the mortar were found to have a negative correlation with the increasing amount of glass sand used.

To investigate the influence of waste glass incorporation in mine tailings backfill on the mechanical properties of the backfill matrix and the underlying mechanisms behind the strength variations after adding glass sand, this study presents a case study on the backfilling of fluorite mine tailings. The main raw materials employed in this investigation include coarse tailings, fine tailings, a cement substitute-curing agent, and recycled waste glass obtained from the mine. Compressive strength changes were assessed by varying the ratios of tailing sand to curing agent and glass dosages. The primary objectives of this study were to evaluate the viability of waste glass in mine tailings backfilling operations and to identify strategies for maximizing the utilization of this resource to promote environmental conservation and sustainable resource management.

## Test material

### Tailings

**Figure 1:**
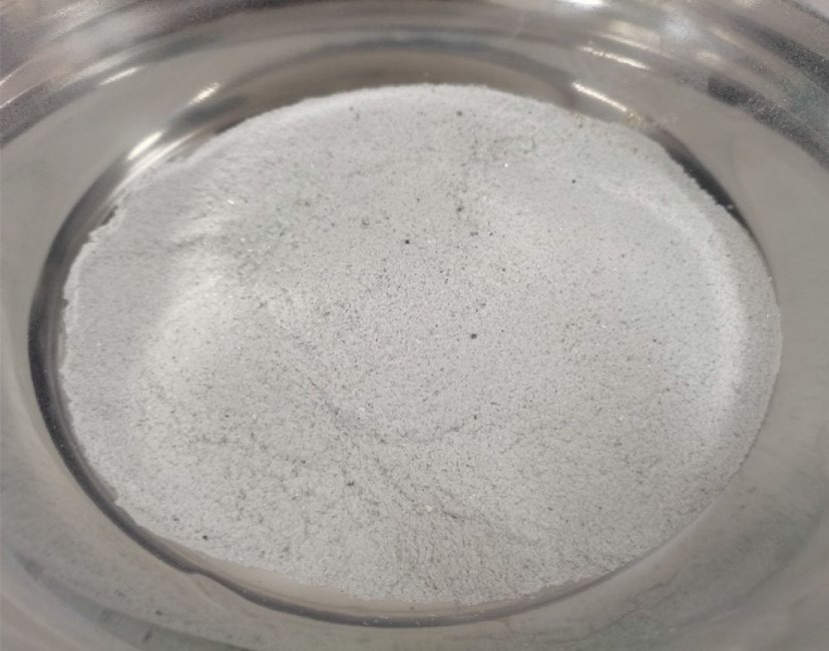
Coarse tailings.

**Figure 2:**
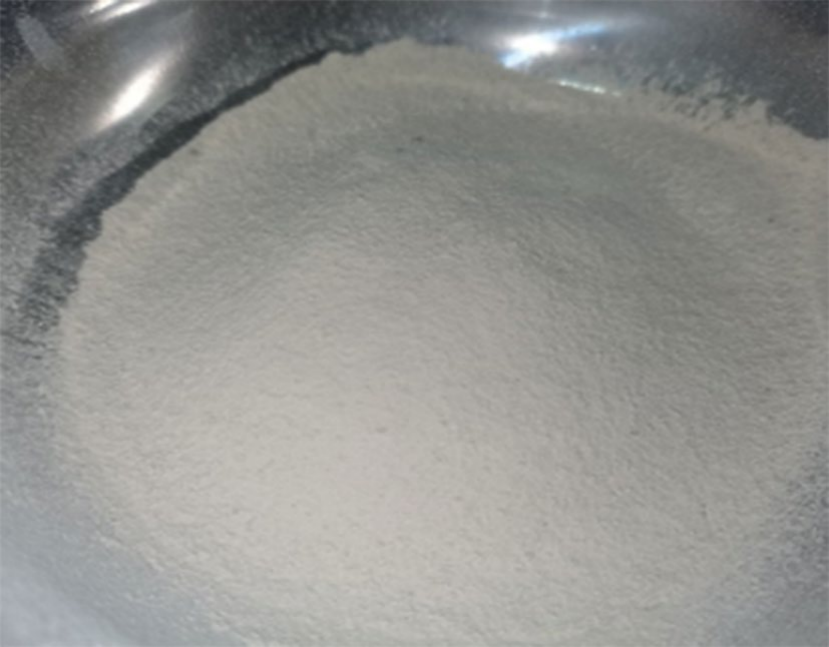
Fine tailings.

The self-generated coarse and fine tailings from this fluorspar mine, utilized for tailings backfill, were employed as the aggregate for the backfill material in the conducted test (Figs. 1, 2). In preparation for the experiment, all samples were subjected to oven-drying, sun-drying, and thorough homogenization. Before testing, the tailings were allowed to undergo natural drying in the laboratory.

To ensure the representativeness of the test results, the quartile method was employed to sample the tailing sand for physical property measurement tests. The physical properties of the tailing sand play a pivotal role in determining the performance of the backfill material. By conducting multiple measurements at various locations, representative data were obtained. The measurement results of commonly utilized physical properties are presented in Table 1.

**Table 1:** Basic physical property parameters.

Material	Densities/(g/cm^3^)	Apparent density/(g/cm^3^)	Packing density /(g/cm^3^)	Dense density /(g/cm^3^)	Porosity/(%)	Specific surface area/(m^2^/g)
Fine tailings	2.659	2.592	1.002	1.310	2.52	1.010
Coarse tailings	2.714	2.615	1.358	1.647	3.65	0.055
Glass sand	2.619	2.506	1.474	1.793	4.31	0.132
Curing agent	3.091	2.904	0.908	1.255	6.05	0.920

**Figure 3:**
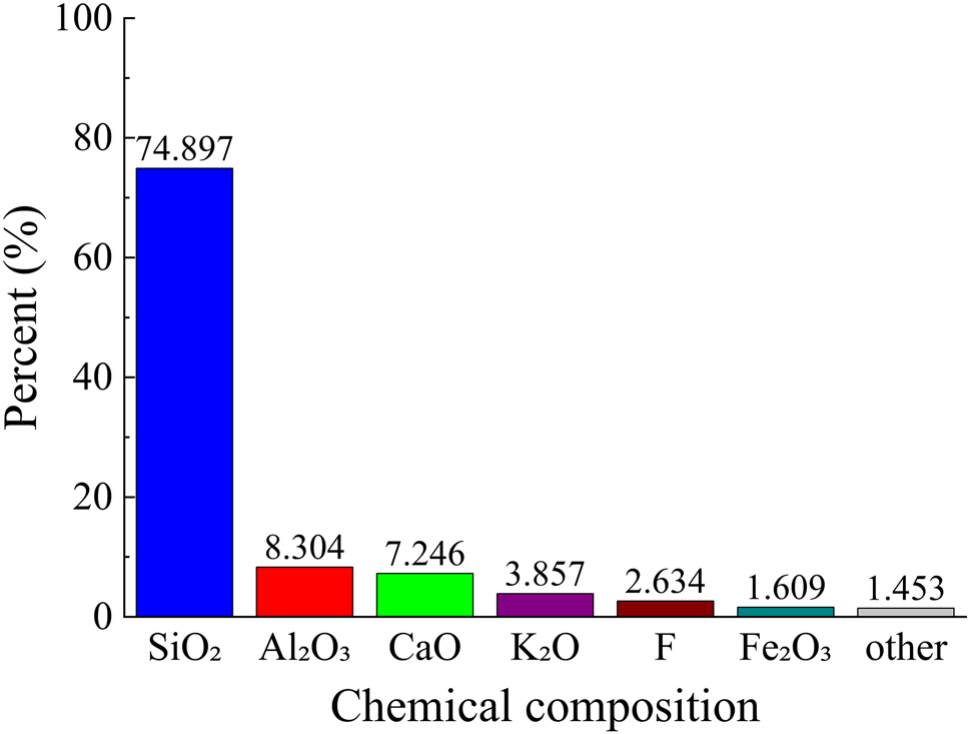
Chemical composition of coarse tailings.

**Figure 4:**
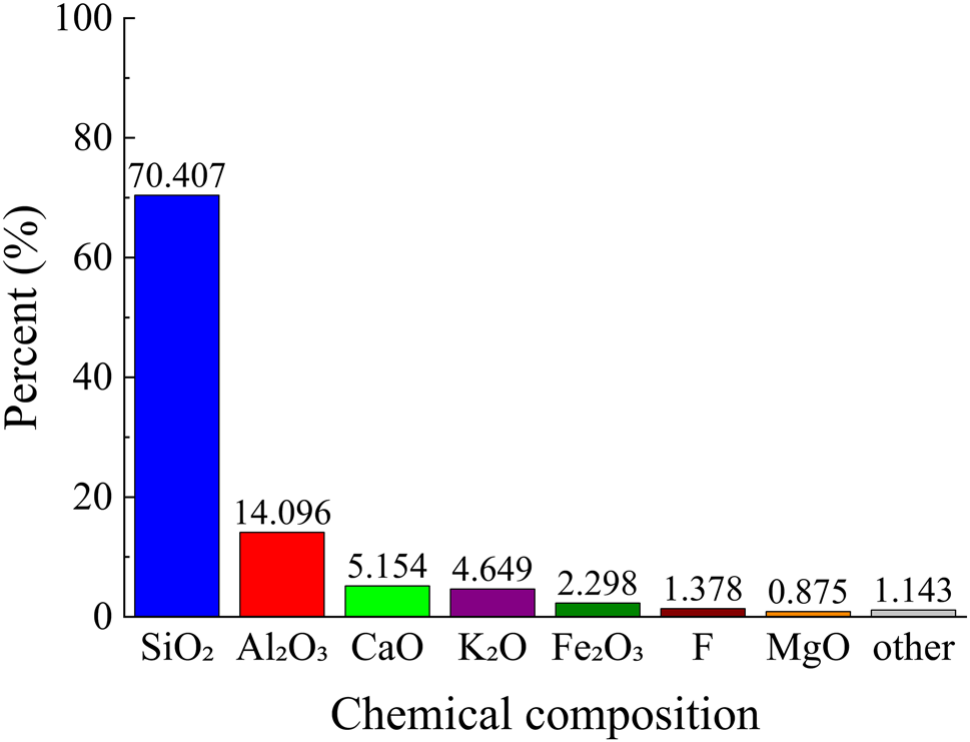
Chemical composition of fine tailings.

The chemical composition of the tailings is presented in Figs. 3, 4, utilizing an Axios wavelength dispersive X-fluorescence spectrometer for analysis. The XRF fluorescence full-scan rapid method was employed as the detection technique at a controlled temperature of 25°°C and relative humidity of 65%. From Figs. 3, 4, it is evident that silica constitutes the primary chemical component in both coarse and fine tailings, with a minor presence of fluorine. However, considering the specific nature of the mine as a fluorite mine where calcium fluoride serves as the principal chemical constituent, it can be concluded that slurry derived from this test material will not generate any additional toxic or hazardous substances during underground backfilling.

**Figure 5:**
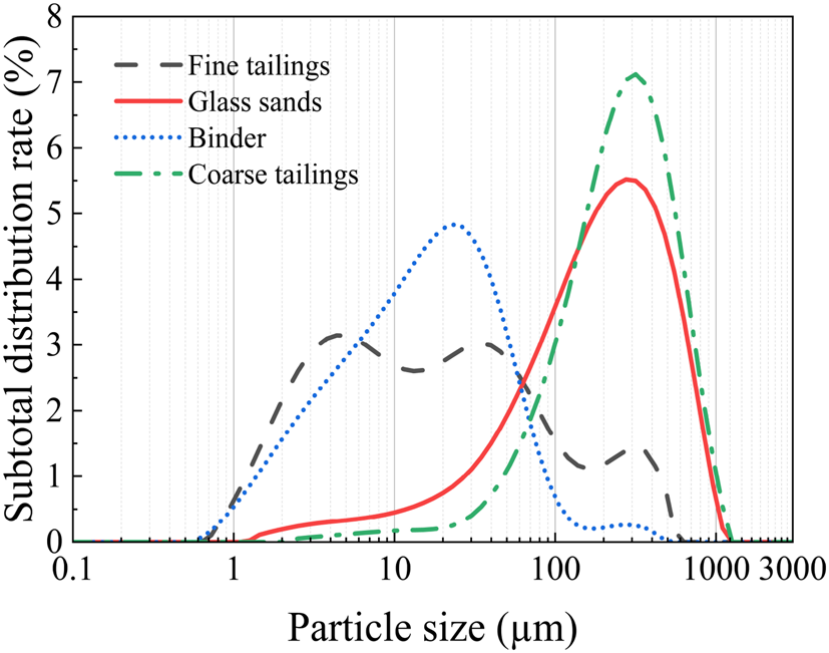
Particle size distribution.

**Figure 6:**
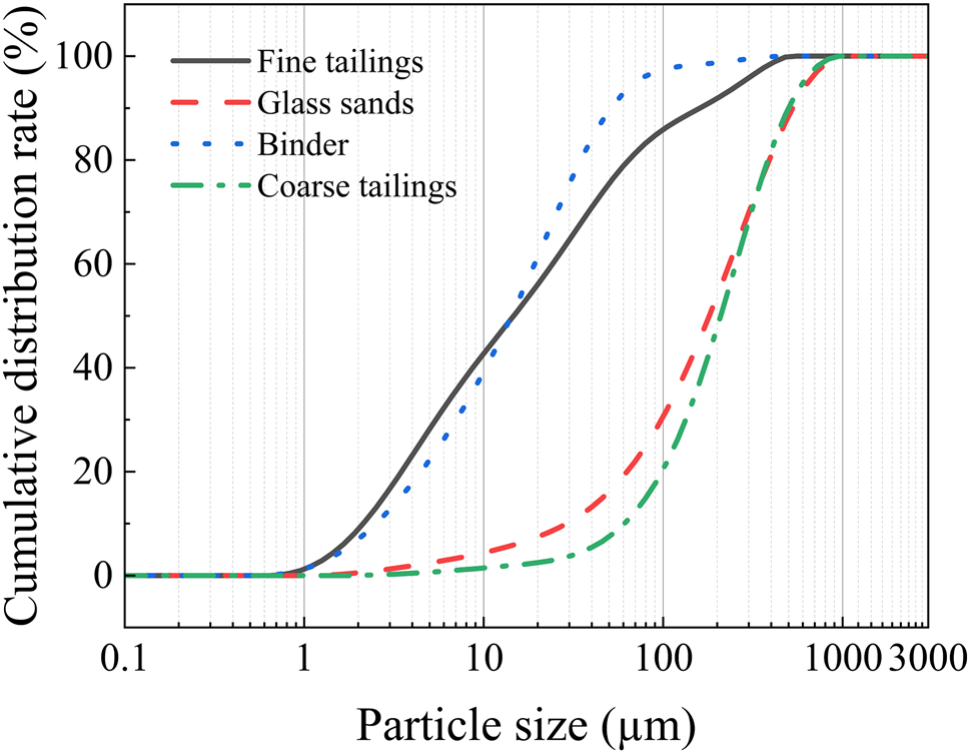
Cumulative distribution ratio of particle size.

The particle size distribution of the tailings was measured using a Malvern 2000 laser particle size analyzer after homogeneously mixing the two tailings in separate containers. The resulting particle size distribution and cumulative particle size distribution curve are presented in Figs. 5, 6. As depicted in Figs. 5, 6, both test materials exhibit fine particle sizes. To ensure long-term prevention of stratification and segregation, as well as the formation of "structural flow" within the backfill slurry pipeline, it is necessary to utilize a significantly higher proportion (greater than 15%) of tailings with a particle size smaller than 20 μm for backfilling purposes, thereby ensuring stable transportation of the backfill slurry^28^.

### Glass sand

**Figure 7:**
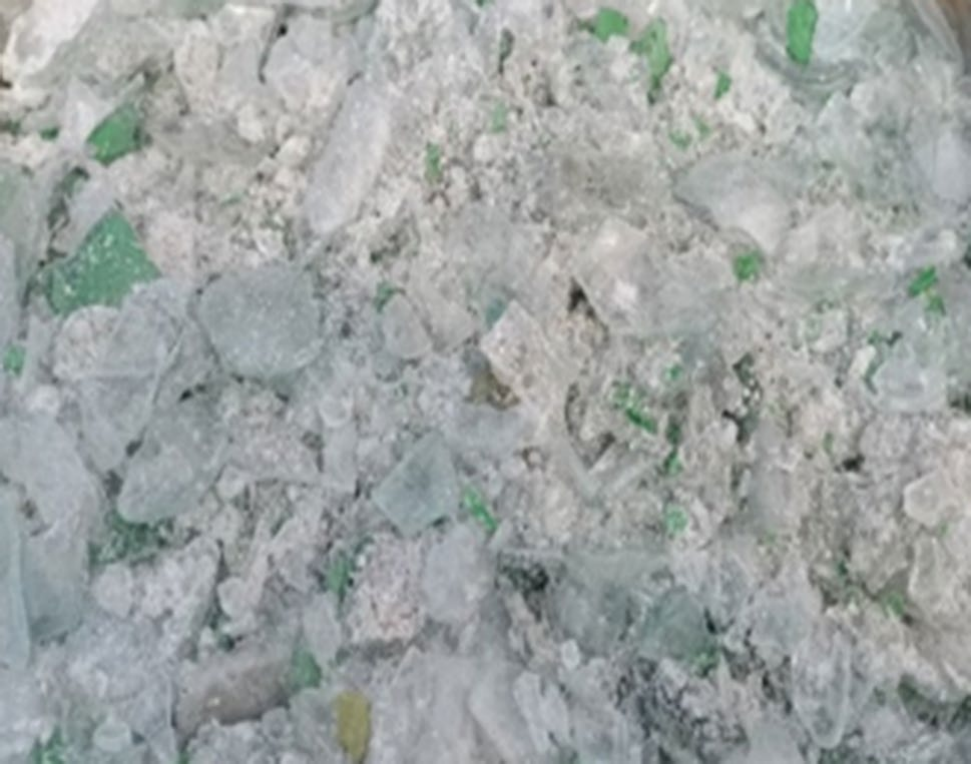
Waste glass after washing and drying.

**Figure 8:**
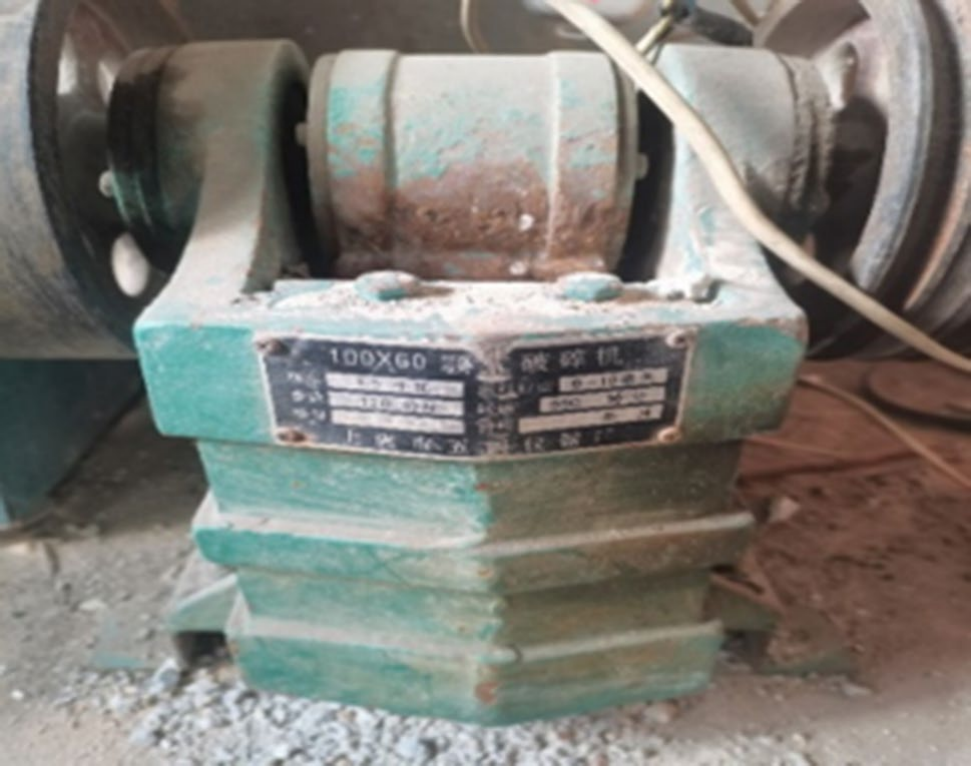
100 × 60 Jaw Crusher.

**Figure 9:**
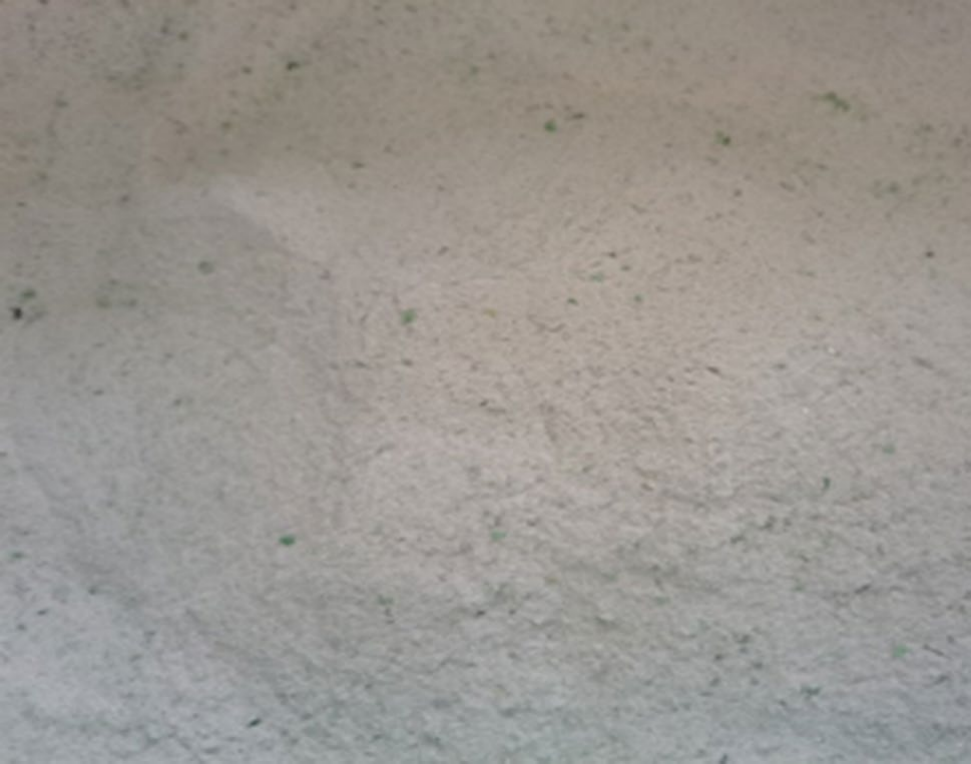
Finished glass sand.

The waste glass underwent a series of processes at the scrap station to produce the finished product with a particle size of less than 1 mm. Firstly, it underwent cleaning and drying (as depicted in Fig. 7). Then, it was crushed using a 100 × 60 jaw crusher (as shown in Fig. 8) and pulverized by Huabus multifunctional pulverizer. And then, it went through screening to achieve the desired particle size distribution (shown in Fig. 9), while measurements of commonly-used physical properties are presented in Table 1.

**Figure 10:**
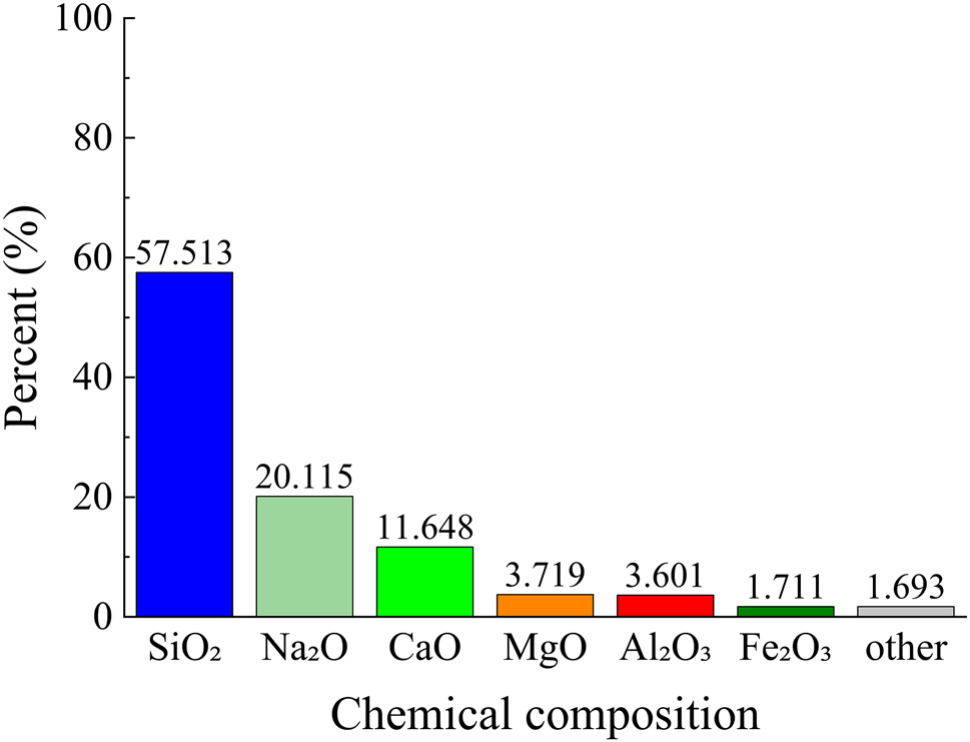
Chemical composition of glass sand.

Due to the intricate composition of the glass sand utilized, in order to gain a more comprehensive understanding of its mechanism for mine tailings backfilling, it is imperative to ascertain the chemical composition of the glass sand as depicted in Fig. 10. By referring to Figs. 3, 4, and 10, it becomes evident that silica constitutes the primary chemical composition of both glass sand and its coarse and fine tailings counterparts. Consequently, employing glass sand for mine tailings backfill materials based on their physical properties and chemical composition appears viable; however, further research remains indispensable.

### Cement material

**Figure 11:**
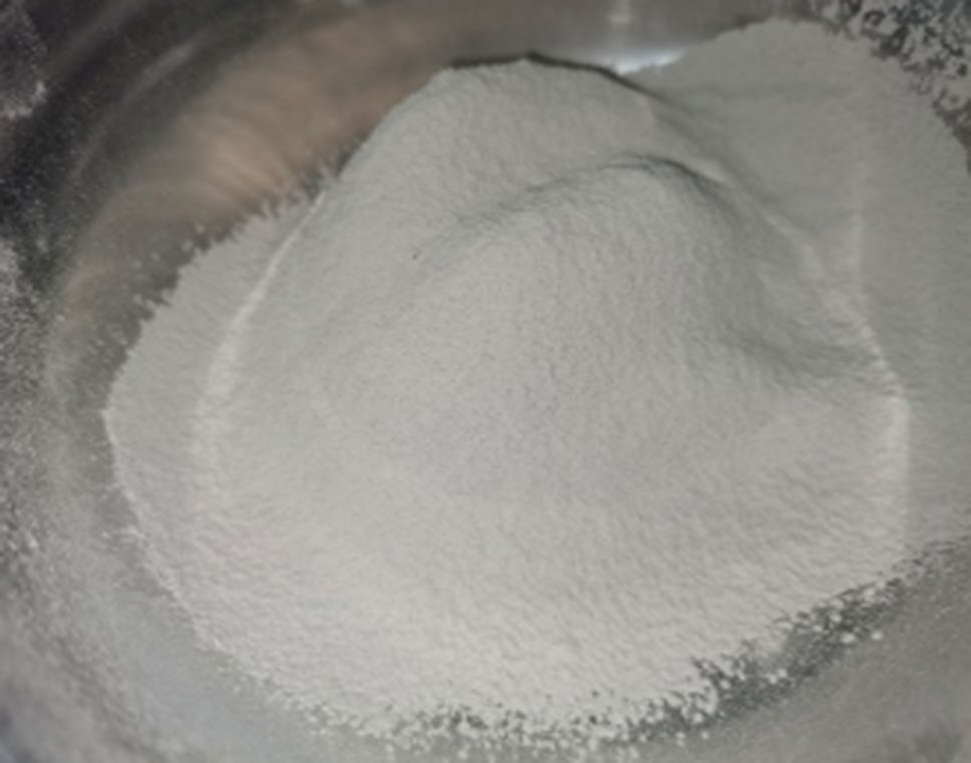
Binder.

**Figure 12:**
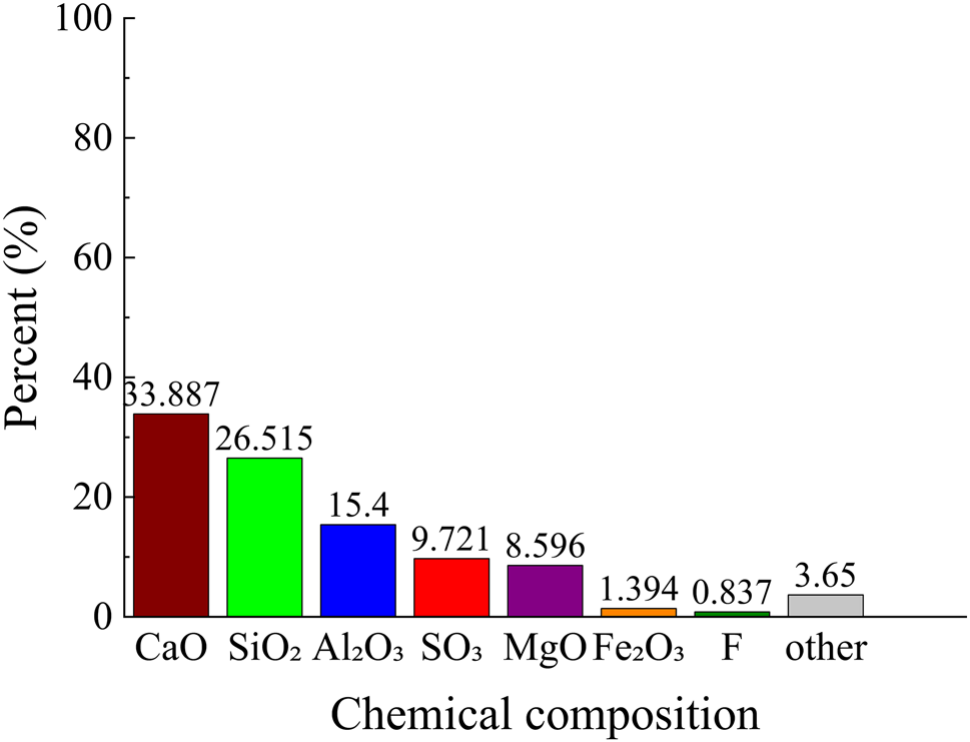
Binder chemical composition.

**Figure 13:**
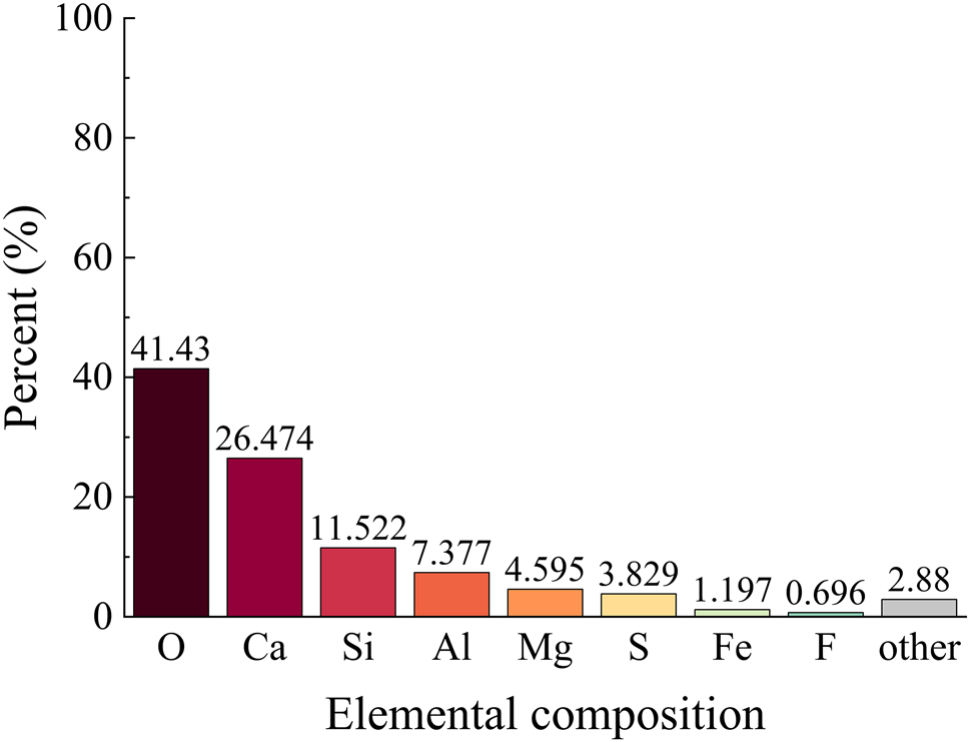
Main monomers composition of the binder.

In the proportioning tests, the binder utilized for mine backfill at this fluorite mine was employed as both the backfill and cementitious material. Following its sampling (as depicted in Fig. 11), a series of tests were conducted to ascertain the physical properties of the binder (as presented in Table 1). The test results pertaining to the primary chemical composition of the binder and the composition of its main monomers are illustrated in Figs. 12, 13. The particle size distribution along with the cumulative distribution rate of particle size are displayed in Figs. 5, 6.

### Water

The test water used in this study was the tailing water discharged from the backfill station of the fluorite mine. Following a comprehensive performance evaluation, it was determined that the density of the tailing water averaged at approximately 1 g/cm^3^, while its pH value measured around 7.1, indicating neutrality. Moreover, during testing, the water temperature ranged between 18 and 22 °C.

## Design with ratio

In order to investigate the impact of incorporating waste glass into tailings backfill on the strength of the backfill body, a more cost-effective approach is adopted. The backfill primarily comprises self-produced tailings sand from the fluorite mine's backfill station, with a slurry concentration of approximately 68%. To ensure more significant test results, we have set the slurry concentration for this experiment at 77%, while maintaining a ratio of 2:1 between coarse and fine tailings sand in the backfill. Under these conditions, four different sand-binder ratios (4:1, 8:1, 12:1, and 16:1) are designed along with six glass dosages (5%, 10%, 15%, 20%, 25%, and 30%) for each group. Additionally, each experimental set includes a control group. The size of the backfill specimens used in testing measures at dimensions of 70.7 mm × 70.7 mm × 70. 7 mm.The specimens with in each group are numbered and tested for strength after ages -3 days,7 days, and 28 days- under standard constant temperature and humidity curing conditions. Three specimens are tested per age category, and their average values are recorded. Tabulated data illustrating suitable sand-binder ratios can be found in Table 2.

**Table 2:** Design with ratio.

Number	Glass dosage/(%)	Coarse tailings/(g)	Glass dosage/(g)	Fine tailings/(g)	Curing agent/(g)	Water/(g)
G0-4	0	410.7	0	205.3	154	230
G5-4	5	390.1	20.5	205.3	154	230
G10-4	10	369.6	41.1	205.3	154	230
G15-4	15	349.1	61.6	205.3	154	230
G20-4	20	328.5	82.1	205.3	154	230
G25-4	25	308	102.7	205.3	154	230
G30-4	30	287.5	123.2	205.3	154	230
G0-8	0	456.3	0	228.1	85.6	230
G5-8	5	433.5	22.8	228.1	85.6	230
G10-8	10	410.7	45.6	228.1	85.6	230
G15-8	15	387.9	68.4	228.1	85.6	230
G20-8	20	365	91.3	228.1	85.6	230
G25-8	25	342.2	114.1	228.1	85.6	230
G30-8	30	319.4	136.9	228.1	85.6	230
G0-12	0	473.8	0	236.9	59.2	230
G5-12	5	450.2	23.7	236.9	59.2	230
G10-12	10	426.5	47.4	236.9	59.2	230
G15-12	15	402.8	71.1	236.9	59.2	230
G20-12	20	379.1	94.8	236.9	59.2	230
G25-12	25	355.4	118.5	236.9	59.2	230
G30-12	30	331.7	142.2	236.9	59.2	230
G0-16	0	483.1	0	241.6	45.3	230
G5-16	5	459	24.2	241.6	45.3	230
G10-16	10	434.8	48.3	241.6	45.3	230
G15-16	15	410.7	72.5	241.6	45.3	230
G20-16	20	386.5	96.6	241.6	45.3	230
G25-16	25	362.4	120.8	241.6	45.3	230
G30-16	30	338.2	144.9	241.6	45.3	230

## Results and discussion

**Figure 14:**
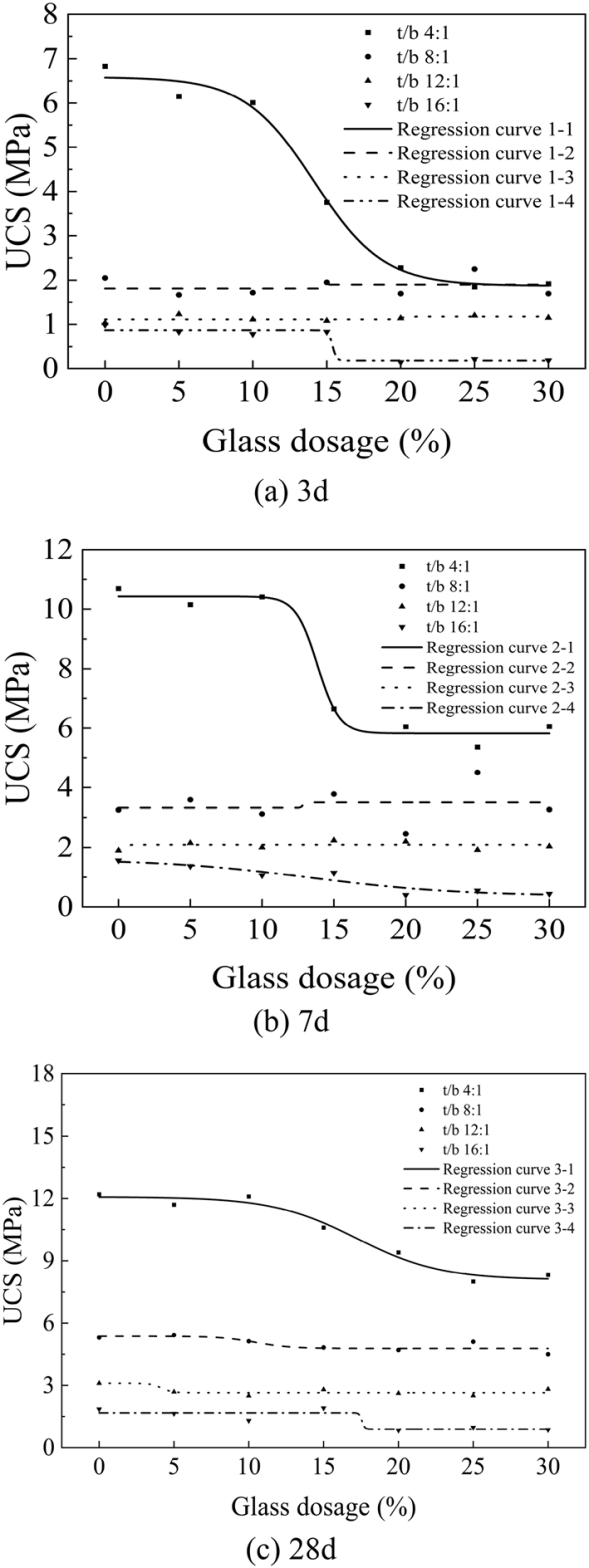
Change rule of USC with sand-binder ratio and glass doping in different age groups.

The DYE-100 electro-hydraulic pressure tester^29^ was utilized to determine the compressive strength of the test block. Subsequently, the obtained test data were analyzed and studied to ascertain the uniaxial compressive strength of the test block at 3 days, 7 days, and 28 days under different sand-binder ratios and various glass admixtures. These results are illustrated in Fig. 14. To depict their change pattern accurately, Boltzmann's function model (Eq. 1) was employed for multiple regression analysis along with establishing multiple regression equations (Table 3). The statistical analysis of the multiple regression model is presented in Table 4^30^.

**Table 3:** Multiple regression equations for USC of test blocks at different sand-binder ratio and at different ages.

Curve number	Regression models
1-1	y_1-1_ = 1.87674 + (6.58447 − 1.87674)/(1 + exp((x − 14.12663)/2.33141))
1-2	y = 1.9 + (1.8133 − 1.9)/(1 + exp((x − 14.6114)/0.02528))
1-3	y = 1.685 + (1.975 − 1.685)/(1 + exp((x − 20.11384)/0.15831))
1-4	y = 0.17667 + (0.86667 − 0.17667)/(1 + exp((x − 15.3896)/0.13527))
2-1	y = 5.82147 + (10.42775 − 5.82147)/(1 + exp((x − 13.85134)/0.7574))
2-2	y = 3.50056 + (3.32156 − 3.50056)/(1 + exp((x − 12.83684)/0.0697))
2-3	y = 1.51732 + (2.09167 − 1.51732)/(1 + exp((x − 0.14506)/0.21274))
2-4	y = 0.36193 + (1.61416 − 0.36193)/(1 + exp((x − 13.33531)/5.22828))
3-1	y = 8.16 + (11.65 − 8.16)/(1 + exp((x − 20.237)/0.18292))
3-2	y = 4.77638 + (5.36136 − 4.77638)/(1 + exp((x − 10.4598)/1.17946))
3-3	y = 2.65 + (3.1 − 2.65)/(1 + exp((x − 4.16285)/0.35971))
3-4	y = 0.88667 + (1.6725 − 0.88667)/(1 + exp((x − 17.56807)/0.09231))

**Table 4:** Regression model statistics.

Curve number	Regression model coefficient error				Reduced Chi-Sqr.	R^2^	Adjusted R^2^
	A_1_	A_2_	X_0_	dx			
1-1	± 0.21342	± 0.18813	± 0.56482	± 0.564764	0.06883	0.99277	0.98554
1-2	± 0.17962	± 0.17959	± 4.7270E5	± 0	0.09676	0.99959	0.99917
1-3	± 0.16276	± 0.11509	± 0.65775	± 0	0.05298	0.99977	0.99955
1-4	± 0	± 0	± 0	± 0	0	1	1
2-1	± 0.27771	± 0.22691	± 3.01236	± 1.95435	0.15394	0.98642	0.97284
2-2	± 0.51042	± 0.44203	± 0	± 0	0.78158	0.99666	0.99332
2-3	± 0.06582	± 0	± 0.32086	± 0	0.02599	0.99989	0.99978
2-4	± 0.46206	± 0.33812	± 4.95029	± 5.65528	0.04737	0.9998	0.99959
3-1	± 0.37207	± 0.52618	± 0.78526	± 0	0.55373	0.99769	0.99537
3-2	± 0.19679	± 0.14677	± 3.3147	± 6.4611	0.06529	0.99972	0.99944
3-3	± 0.1774	± 0.0794	± 1.9053	± 0	0.03147	0.99987	0.99973
3-4	± 0.13963	± 0.16123	± 0	± 0	0.07798	0.99967	0.99933

1 $${Y}_{a}=\frac{{A}_{1}-{A}_{2}}{1+{e}^{(x-{x}_{0})/dx}}+{\text{A}}_{2}$$

The coefficient error and Reduced Chi-Sqr of the regression model are important indicators for assessing the predictive accuracy, stability and interpretability of the model, and larger values indicate. The goodness of fit is usually expressed by R^2^, whose value ranges from 0 to 1. The closer R^2^ is to 1, the better the model fits the observed data, i.e., the model is able to explain more of the variability of the data; the closer R^2^ is to 0, the worse the model fits the observed data, i.e., the model is unable to explain the variability of the data well.

The multiple regression equations presented in Table 3, the Boltzmann function in the given form demonstrates that $Y=\frac{{A}_{1}-{A}_{2}}{1+{e}^{(x-{x}_{0})/dx}}+{\text{A}}_{2}$ is used and the iterative algorithm of orthogonal distance regression is used to fit the change rule of uniaxial compressive strength of specimens under four different sand-cement ratios with different glass doping, The regression curves 1-4 exhibit a significant increase in magnitude solely due to the sudden decline in strength, while their corresponding R^2^ values for fitting are below 0.99 and 0.96 respectively; however, all other curves have R^2^ values exceeding 0.99, This indicates that the fitted curves were fitted with high precision, predicted their intensity development with high accuracy, and were able to explain more of the data variability..The coefficients of the fitting curves in Table 4 demonstrate a positive correlation between age and both the parameters and their range of values. Notably, when the ratios of tailings to curing agent are at 8:1 and 12:1, the fluctuation ranges of parameter values for fitting curves 1-2, 1-3, 2-2, 2-3, 3-2, and 3-3 are smaller. This finding aligns with the conclusion that compressive strength fluctuations increase with sand-binder ratio and glass mixing amounts. Furthermore, it supports the observation that higher ratios of tailings to hardener at varying glass dosages lead to increased compressive strength fluctuations at different ages.

The compressive strength of the test block at a 3-day age decreases with an increase in glass dosage, as illustrated in Fig. 14a for a sand-binder ratio of 4:1. This can be attributed to the presence of a significant amount of Na_2_O in the glassy sand, which reacts with water to produce a substantial quantity of NaOH. Consequently, an alkali aggregate reaction similar to that observed in concrete occurs, leading to a sudden decline in the strength of the test block. The 3-day compressive strength of the sand-binder ratio of 4:1 is lower than that of the sand-binder ratio of 8:1 when a glass admixture is added at a concentration of 25%. When the sand-to-binder ratio is 4:1, 8:1, 12:1, and 16:1 with glass doping less than 15%, the effect of glass doping on compressive strength shows slight fluctuations compared to the control group. The range of fluctuation is observed as follows: 5–18%, 5–19%, and 16–21% respectively. Moreover, when glass doping reaches a level of 25% with a sand-ash ratio of 8:1, early strength characteristics are exhibited in terms of compressive strength. In this case, the strength surpasses that achieved with a sand-to-binder ratio of 4:1 by an increase of approximately 0.4 MPa due to chemical reactions between waste glass mixed into backfill slurry and curing agents which generate silica gel similar to crystals. Consequently, these reactions lead to decreased hydroxide ion concentration in specimens while promoting hydration reaction and enhancing both overall strength and early strength properties. However, using a sand-binder ratio of 16:1 combined with glass dosage exceeding 15% results in an abrupt decrease in compressive strength ranging from 0.62 to 0.69 MPa.

From Fig. 14b, it can be observed that, for varying sand-binder ratios, the influence of glass dosage on the 7-day compressive strength increases as the sand-binder ratio increases. The impact of tailings to curing agent ratios (4:1, 8:1, 12:1, and 16:1) on compressive strength ranges from 1 to 5%, from 4 to 10%, from 10 to 13%, and from 13 to 32.5%. When the sand-to-binder ratio is 8:1 or 12:1 and the glass admixture is less than 15% but more than 10%, it enhances the compressive strength of specimens by 0.53 MPa and 0.34 MPa, respectively. However, when the sand-to-binder ratio is at a lower level of 4:1 and glass admixture exceed 10%, it results in a significant decrease in compressive strength with a magnitude of about -3.76 MPa, followed by slight recovery at a glass dosage of around 30% (with an improvement value of approximately + 0.69 MPa). This phenomenon can be attributed to the active volcanic ash activity present in glass powder that fills tiny voids within concrete particles, forming structures similar to those found in high-solid-content concrete mixtures that enhance mechanical properties within backfill bodies^31^. Additionally, when using a sand-to-binder ratio of either 8:1 or adding varying amounts (5%,15%,25%) of glass admixture, early strength characteristics are still observed with increased strengths ranging from + 0.3 to + 1.3 MPa compared to control groups.

As shown in Fig. 14c, the 28-day compressive strength exhibits a stepwise decrease, with a diminishing amplitude of 1.5 MPa and 2.9 MPa respectively, when the sand-binder ratio is small (4:1) and the glass doping exceeds 10%. The influence of various glass doping on the compressive strength is enhanced as the sand-binder ratio increases, particularly when it reaches high ratios such as 8:1, 12:1, and 16:1. The fluctuation range is 2.26–15.09%, 9.03–19.35%, and 2.70–55.14%. It should be noted that the disappearance of early strength characteristics at a glass doping level of 25% can be attributed to the gradual formation and growth of "cement stone" over time, leading to a gradual weakening effect on waste glass particles. The formation and growth of the "cement stone" over time leads to a gradual weakening of the effectiveness of waste glass particles.

## Micro-mechanism analysis

**Figure 15:**
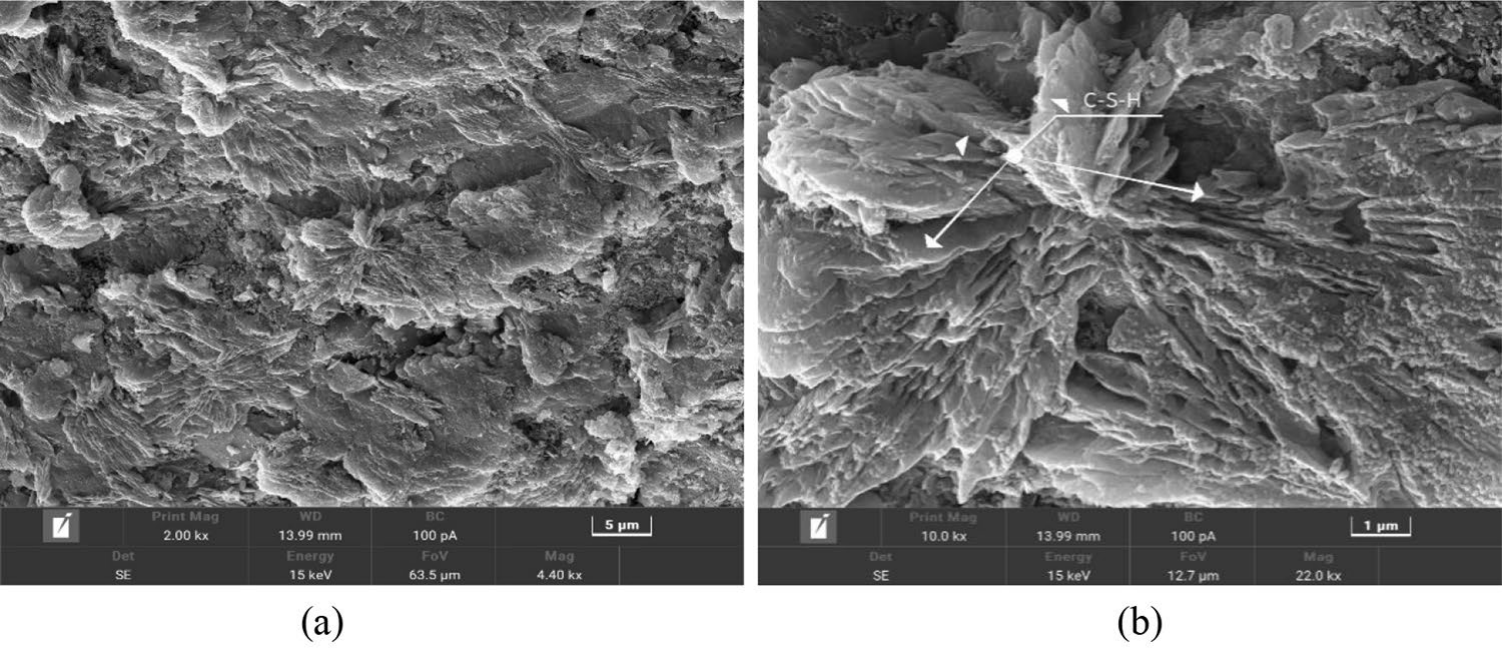
glass doping 0%.

**Figure 16:**
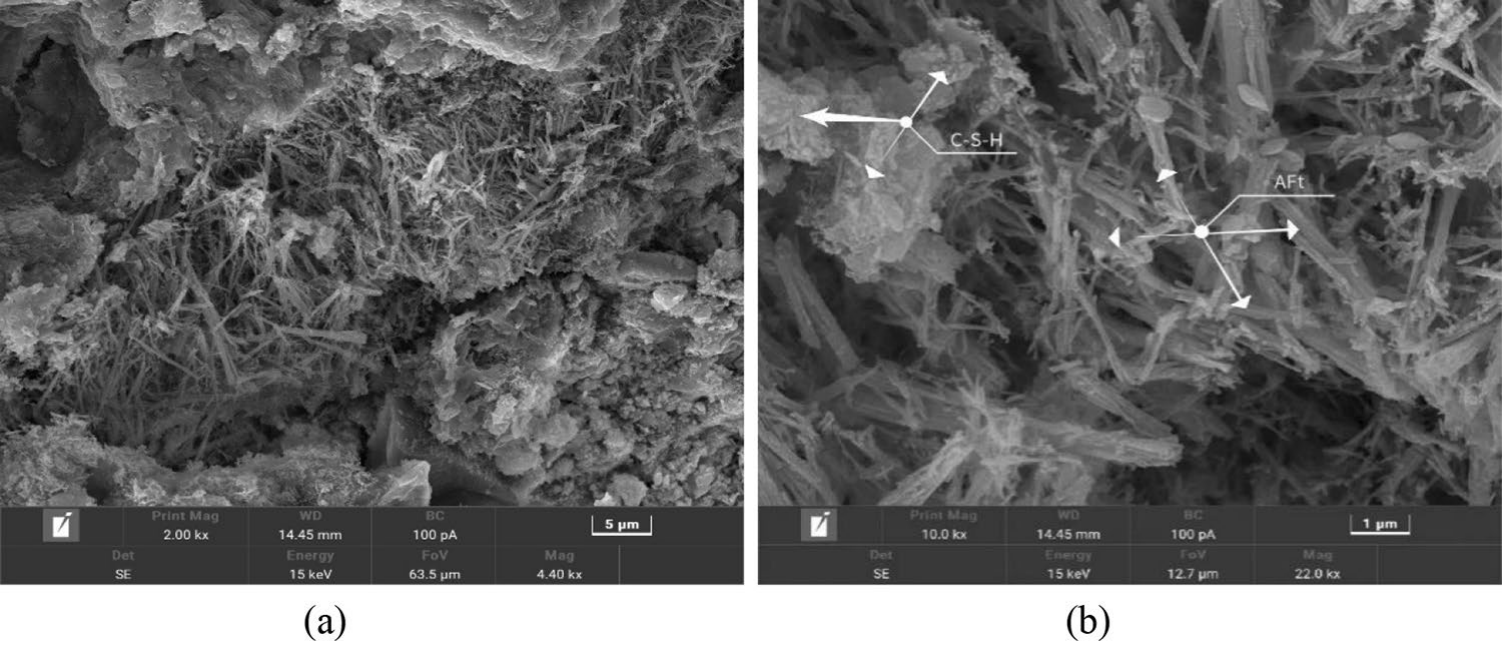
glass dosing 25%.

To further investigate the cause of the sudden decrease in specimen strength with increasing glass sand doping at low sand-binder ratios, microphotographs were taken using a Czech TESCAN MIRA LMS electron microscope. Two sets of SEM images were obtained, capturing specimens at 2 K and 10 K magnifications, as depicted in Figs. 15 and 16.

As illustrated in Fig. 15, due to the close chemical composition between the curing agent and cement, the latter exhibits cementitious characteristics during the hydration reaction of specimen blocks. Consequently, numerous agglomerates form clusters of hydrated calcium silicate gel (C-S-H) within the blocks without glass sand addition. This phenomenon effectively fills up internal pore spaces and results in a more densely packed structure that promotes backfill body strength development^32^. The main chemical composition of glass sand in Figs. 10 and 15 reveals a significant presence of Na_2_O, which leads to the generation of a substantial amount of NaOH when combined with water during the early strength formation of the test block. Consequently, this results in an increase in OH^−^ concentration within the solution, thereby impeding the solubilization process of Al(OH)^−4^, Ca^2+^, and SO_4_^2+^ present in caliche^33^, The OH^−^ ions will trigger the active SiO_2_ Alkali Aggregate Reaction (ARR) by reacting with the formed calcium silicate gel, resulting in the formation of numerous intertwined acicular, rod-shaped, prismatic, and radial calcium alunite crystals (AFt). Additionally, a small amount of C-S-H gel will interlace with these crystals to fill the internal pores of the test block^33–35^, The rate of early hydration reaction in the test block increases, leading to internal expansion or even cracking. When the doping of glassy sand is less than 15%, specifically Na_2_O doping is lower, these phenomena occur. However, when the dosage of glass sand exceeds 25% and there is an increase in Na_2_O dosage, the internal structure of the specimen expands, resulting in a sudden decrease in its strength. This verifies the analysis explaining why there is a sudden drop in strength when the ratio of tailing sand to curing agent is 4:1 and the glass dosage is 25%.

## Conclusion

In this experiment, 216 specimen samples were tested to investigate the effects of sand-cement ratio, glass doping and age on the compressive strength of specimens, and the corresponding strength prediction model was fitted, whereas the mechanism of the action of waste glass in tailings filling was derived through the micro-mechanism analysis and the following conclusions were obtained:

1. The incorporation of an appropriate amount of waste glass particles in the backfill body can enhance its mechanical properties and early strength characteristics. The compressive strength of the specimen is influenced by variations in tailings and curing agent ratios, as well as different glass admixtures. Moreover, the increase in sand-binder ratio and aging duration also affects the fluctuation amplitude of compressive strength.
2. The influence of waste glass on the late stage of the backfill body is found to be minimal in sand-binder ratios of 8:1 and 12:1, further confirming the feasibility of utilizing waste glass for mine tailing backfill. In this test with a mass concentration of 77% and a coarse tailings to fine tailings ratio of 2:1, the optimal sand-binder ratio for waste glass used in mine tailings backfill is determined as 8:1, with a recommended mixing proportion of glass at 25%, this is also in line with the conclusions reached in related studies^16,19^.
3. After conducting strength analysis and SEM microanalysis on the specimen with a lower sand-binder ratio, it is recommended that the dosage of glass sand should not exceed 10% when the tailings to curing agent ratio is low. Otherwise, an excessive amount of glass sand may cause violent expansion within the specimen, thereby adversely affecting its strength formation.

Overall, this paper innovatively uses waste glass as tailings filling material, which is an innovation and breakthrough to the traditional tailings filling material. Through research and analysis, an effective method is found to use waste glass for tailings filling in order to solve the problem of environmental protection and resource recycling of tailings and waste glass. The study also aims to promote the progress of scientific research and provide more theoretical basis and research methods in this field.

## Data Availability

All data generated or analysed during this study are included in this published article.
